# PME aiming for quality, originality and peer recognition

**DOI:** 10.1007/s40037-012-0015-2

**Published:** 2012-05-22

**Authors:** Griet Peeraer

**Affiliations:** Faculty of Medicine and Health Sciences, University of Antwerp, Antwerp, Belgium

When asked about criteria for assessing quality in academic research, 94 scientists working in the domain of health research but coming from different backgrounds (biomedicine, medicine and social sciences) believed that peer-reviewed articles were still the best academic achievement indicators [1].

According to Albert’s study, the three interrelated arguments that were emphasized when talking about peer-reviewed published articles were quality, originality, and peer recognition of the studies.

## In this issue of PME

PME is a peer-reviewed journal and in my view the same three arguments should be considered when reading the manuscripts submitted to PME. In Wingelaar’s article [2] on student’s educational needs for clinical reasoning in first clerkships, we note that the results of this study underscore the importance of learning in a clinical setting. Intrinsic motivation for learning is enhanced when students take care of *real* patients; they feel a stronger need to learn how to perform effectively compared with being trained in a simulated situation.

According to literature on self-efficacy, however, most students overestimate their capabilities. According to Artino [3], medical students who overestimate their self-efficacy may possess high confidence in their ability to draw blood after having learnt the skill, but yet may fail to draw blood correctly from a patient in a clinical setting.

The two articles are both original, of good quality and have been recognized by peers. They provide interesting information and a challenge for further research. The information given by PME articles will hopefully fuel the debate on simulated learning and workplace-based ‘real life’ learning.

A different debate in medical education research is the one on applying research methods from the clinical research paradigm in education research [4]. Randomized controlled trials (RCT) have traditionally been perceived as the strongest design for research [5]. Salina et al. [6] chose the RCT method to study the effectiveness of YouTube for the learning of a nursing technique and found that video represents an important learning tool to refresh and reinforce supporting student learning. In this technological era, YouTube as a video-sharing website is known to almost everyone. When this and the next generation(s) of students grow up with media in a way totally different from former generations, aspects such as educational approaches, instructional design have to change.

As Sir Ken Robinson stated in his lecture Changing Education Paradigms [7], we are still shaping the future by doing what people did in the past. Therefore, educational research on the use of new media in classrooms should be promoted. There is a need for thorough research on this topic, with research methods adapted to the educational environment.

Other contributions to this PME issue focus on faculty and student perceptions of the feasibility of individual student–faculty meetings [8], on cognitive outcomes in a pre-clinical bioethics course [9] and on the Ph.D. thesis by Dr. K. Overeem [10]. She has developed a new system for doctor performance assessment and found evidence on how we might optimize multisource feedback in improving doctor performance.

## PME and the future

It is in the best interest of medical education and the training of future doctors that research on medical education keeps growing.

Research in medical education is a relatively young domain. It draws upon scientific principles from a variety of domains/disciplines. The different disciplines have shared assumptions about what ‘good’ science is, what methods are best to generate valid results, how data should be collected and interpreted, et cetera [11]. Medical education research is a domain that is open to collaboration and to research methods from other disciplines. PME as a journal will try to reflect this view in the choice of its articles.
